# Using LMS tables to determine waist circumference and waist-to-height ratios in Colombian children and adolescents: the FUPRECOL study

**DOI:** 10.1186/s12887-017-0919-4

**Published:** 2017-07-11

**Authors:** Robinson Ramírez-Vélez, Javier Moreno-Jiménez, Jorge Enrique Correa-Bautista, Javier Martínez-Torres, Katherine González-Ruiz, Emilio González-Jiménez, Jacqueline Schmidt-RioValle, Felipe Lobelo, Antonio Garcia-Hermoso

**Affiliations:** 1 Centro de Estudios para la Medición de la Actividad Física (CEMA). Escuela de Medicina y Ciencias de la Salud, Universidad del Rosario, Cra. 24 No. 63C - 69, Bogotá D.C, Colombia; 2 Grupo de Ejercicio Físico y Deportes, Facultad de Salud, Vicerrectoría de Investigaciones, Universidad Manuela Beltrán, Bogotá D.C, Colombia; 3 Departamento de Enfermería, Facultad de Ciencias de la Salud, Universidad de Granada, Avda. De la Ilustración, s/n, 18016 Granada, Spain; 4 Grupo CTS-436: Centro de Investigación Mente, Cerebro y Comportamiento (CIMCYC), Granada, Spain; 5 Hubert Department of Global Health, Rollins School of Public Health, Emory University, Atlanta, GA USA; 6 Laboratorio de Ciencias de la Actividad Física, el Deporte y la Salud, Facultad de Ciencias Médicas, Universidad de Santiago de Chile, USACH, Santiago, Chile

**Keywords:** Central obesity, Reference values, Anthropometric indices

## Abstract

**Background:**

Waist circumference (WC) and waist-to-height ratio (WHtR) are often used as indices predictive of central obesity. The aims of this study were: 1) to obtain smoothed centile charts and LMS tables for WC and WHtR among Colombian children and adolescents; 2) to evaluate the utility of these parameters as predictors of overweight and obesity.

**Method:**

A cross-sectional study was conducted of a sample population of 7954 healthy Colombian schoolchildren [3460 boys and 4494 girls, mean age 12.8 (±2.3) years]. Weight, height, body mass index (BMI), WC and WHtR were measured, and percentiles were calculated using the LMS method (Box-Cox, median and coefficient of variation). Appropriate cut-off points of WC and WHtR for overweight and obesity, according to International Obesity Task Force definitions, were selected using receiver operating characteristic (ROC) analysis. The discriminating power of WC and WHtR is expressed as area under the curve (AUC).

**Results:**

Reference values for WC and WHtR are presented. Mean WC increased and WHtR decreased with age for both genders. A moderate positive correlation was observed between WC and BMI (*r* = 0.756, *P* < 0.01) and between WHtR and BMI (*r* = 0.604, *P* < 0.01). ROC analysis revealed strong discrimination power in the identification of overweight and obesity for both measures in our sample population. Overall, WHtR was a slightly better predictor of overweight/obesity (AUC 95% CI 0.868–0.916) than WC (AUC 95% CI 0.862–0.904).

**Conclusion:**

This paper presents the first sex and age-specific WC and WHtR percentiles for Colombian children and adolescents aged 9.0–17.9 years. The LMS tables obtained, based on Colombian reference data, can be used as quantitative tools for the study of obesity and its comorbidities.

## Background

The prevalence of overweight and obesity is a global public health problem [1]. Recesntly studies indicate that increased body weight and inadequate body fat distribution are associated with adverse health problems, including hypertension, cardiovascular disease, metabolic disorders, and asthma, as well as multiple malignancies [2–4]. International organisations and epidemiological cross-sectional studies have suggested that individuals with a hight porcentage of body fat in the abdominal region are at greater risk of developing metabolic syndrome [5–9]. Body fat in general and abdominal fat in particular are often studied using anthropometric indicators such as waist circumference (WC), waist-to-height ratio (WHtR) and body mass index (BMI) [8, 10, 11]. These indicators are simple to measure and calculate, inexpensive and applicable to a large number of individuals in epidemiological studies and clinical practice [10–14].

The two most widely used definitions of abdominal fat are those of the National Cholesterol Education Program Adult Treatment Panel III (NCEP: ATPIII) and of the International Diabetes Federation (IDF). Both focus on WC, as a surrogate measure of central obesity [5, 14]. Another useful anthropometric indicator of body fat deposits is the waist-to-height ratio (WHtR), also termed the index of central obesity [3, 15–17]. Ashwell et al. [10], in a meta-analysis, confirmed that measures of abdominal obesity, especially WHtR, provide a superior tool for discriminating obesity-related cardiometabolic risk, compared with BMI.

Colombia, like other low and middle-income countries in Latin America and also in Africa, is undergoing a situation of nutritional transition, with a growing prevalence of overweight and obesity among the population. Accordingly, criteria should be established to identify populations at high risk of presenting excessive body fat, so that preventive interventions can be designed and implemented [18–20].

Ethnic and environmental differences probably influence body proportions, and so national references would be useful to control for variations between populations. Cut-off values and percentiles for WHtR and WC are available for children and adolescents in various countries [21–26], but not in Colombia. Although studies have been conducted of adolescent populations in Colombia, using LMS methods, and have obtained representative percentiles, by age and sex, for certain anthropometric indicators (percentage of body fat, BMI, WC and waist/hip ratio) [27, 28], these studies were based on small samples, and more extensive studies are needed to better characterise the child and adolescent population of Colombia.

Taking these considerations into account, the aims of this study were to establish smoothed centile charts and LMS tables for WC and WHtR in a population of Colombian children and adolescents and to evaluate the utility of these parameters as predictors of overweight and obesity.

## Methods

### Study design and sample population

This cross-sectional study was conducted among a sample population consisting of healthy Colombian children and adolescents. In this country, data on young people’s weight, height and physical activity, among other parameters, are recorded in public health monitoring systems [29]. In this respect, the FUPRECOL study (*In Spanish*: [Asociación de la fuerza prensil con manifestaciones de riesgo cardiovascular tempranas en niños y adolescentes colombianos], Association between grip strength and early signs of cardiovascular risk in Colombian children and adolescents) [20, 28, 30] was performed to determine levels of physical fitness among children and adolescents in Colombia, and to determine their relation with the general prevalence of cardiovascular risk factors in this population. The FUPRECOL study assessments were conducted during 2014–2015.

The sample in the present study consisted of 7954 healthy Colombian schoolchildren with an average age of 12.7 (± 2.4) years, with 3460 boys and 4494 girls. This sample represented 72.3% of the sample size of the primary FUPRECOL study, which included 11,000 schoolchildren aged from 9 to 14 years, from families with a low socioeconomic status and attending State-funded schools in Bogotá. The present study included only those participants who completed the same tests and were subjected to the same methodological approach for anthropometric variables.

In recruiting the sample, 27 public elementary and high schools (grades 5–11) were selected in the capital district of Bogotá (Cundinamarca Department, Andean region) [31]. These schools were selected taking into account the existence of collaboration agreements with our research centre, and so were selected mainly for pragmatic, budgetary and logistic reasons. Thus, convenience sampling was performed.

All the children in our study population were of low-middle socioeconomic status (1–3 on a scale of 1–6 defined by the Colombian government). The sample was grouped by age in 1-year increments and sex. The significance level was set to 0.05, and the required power was set to at least 0.80. Power calculations were based on the mean values for overweight and obesity among the first 200–400 participants in the ongoing data collection (range: 26–32 kg/m^2^), with a group SD of approximately 5.2 kg/m^2^. Finally, the sample size was calculated to be approximately 250–500 participants per group. Exclusion factors included a clinical diagnosis of cardiovascular disease, diabetes mellitus 1 or 2, pregnancy, the use of alcohol or drugs, and, in general, the presence of any disease not directly associated with nutrition. Exclusion from the study was made effective a posteriori, without the students being aware of their exclusion.

### Measures

Each participant underwent a complete anthropometric evaluation performed according to the International Society for the Advancement of Kinanthropometry [32]. Body weight was measured, using electronic scales (Tanita® BC544, Tokyo, Japan) with a low technical error of measurement (TEM = 0.510%). Height (Ht) was measured using a portable stadiometer (Seca® 274, Hamburg, Germany; TEM = 0.019%). BMI was calculated as weight divided by height squared (kg/m^2^). Waist circumference was measured at the midpoint between the last rib and the iliac crest using a tape measure (Ohaus® 8004-MA, New Jersey, USA; TEM = 0.086%) [20]. WHtR was calculated as the ratio of WC (in cm) to Ht (in cm). Overweight and obesity were defined as BMI above the age and sex-specific thresholds of the IOTF [33]. According to this definition, the group of subjects with overweight (the equivalent of BMI ≥25 kg/m^2^) also contains those who are obese (the equivalent of BMI ≥30 kg/m^2^).

### Statistical analyses

Anthropometric characteristics from the study sample are presented as mean and standard deviation (SD). Normality for selected variables was verified using histograms and Q-Q plots. Data were then split by sex, and a one-way ANOVA with post hoc tests (Tukey) was used to identify differences between age groups within sexes. Smoothed age and gender-specific table percentiles (3rd, 10th, 25th, 50th, 75th, 90th and 97th) were constructed for WC and WHtR via a penalised maximum likelihood approach. The following abbreviations are used: (1) M (median), (2) L (Box–Cox transformation) and (3) S (coefficient of variation). The associations between WC, WHtR and BMI were tested by means of Pearson correlation coefficients. The relations between WC, WHtR and overweight/obesity as defined by IOTF [33] were investigated using receiver operating characteristic (ROC) curves. Cut-off values were derived mathematically from the ROC curves, using the point on the ROC curve with the lowest value for the formula: (1-sensitivity)^2^ + (1-specificity)^2^. The positive likelihood ratio LR (+) and the negative likelihood ratio LR (−) were also determined. Descriptive statistics were calculated with SPSS 21.0 (SPSS Inc. Chicago, IL, USA). Statistical significance was set at *P* < 0.05.

## Results

Descriptive statistics for weight, Ht, BMI, WC and WHtR by age group are presented in Table 1. The corresponding percentiles are listed in Table 2 (WC) and Table 3 (WHtR). Mean BMI values were comparable in both sexes, and the prevalence of overweight was 25.0% (95% CI: 23.5–26.6%) and 15.8% (95% CI: 14.4–17.3 5%) in girls and boys, respectively. The prevalence of obesity was 9.9% (95% CI: 8.9–11.0%) and 7.5% (95% CI: 6.5–8.5%) in girls and boys, respectively. Mean WC increased and WHtR decreased with age for both genders. In both sexes, there was a moderate positive correlation between WC and BMI (*r* = 0.756, *P* < 0.01) and between WHtR and BMI (*r* = 0.604, *P* < 0.01).

**Table 1:** Mean values (standard deviation, SD) for body weight (BW), height (Ht), body mass index (BMI), waist circumference (WC) and waist-to-height ratio (WHtR) for Colombian children and adolescents aged 9–17.9 years

Sex	n	Body weight (kg)	Height (cm)	BMI (kg/m^2^)	WC (cm)	WHtR
Boys						
9 to 9.9	258	32.1 (7.5)	133.5 (6.5)	17.8 (3.1)	60.8 (6.7)*	0.455 (0.044)**
10 to 10.9	466	34.5 (8.5)	137.3 (7.4)*	18.1 (3.3)	61.7 (8.0)*	0.450 (0.052)**
11 to 11.9	445	37.2 (8.8)*	141.9 (8.2)*	18.3 (3.2)	63.5 (7.6)*	0.448 (0.047)**
12 to 12.9	404	41.3 (9.1)*	147.1 (8.2)*	18.9 (3.2)	64.7 (7.5)*	0.440 (0.047)**
13 to 13.9	401	46.0 (9.8)*	153.5 (9.3)*	19.4 (3.3)**	65.8 (7.7)	0.429 (0.048)
14 to 14.9	443	50.0 (9.7)*	158.9 (9.1)**	19.7 (3.0)**	67.1 (7.1)	0.423 (0.043)**
15 to 15.9	426	54.4 (9.7)*	163.3 (8.9)**	20.3 (3.0)**	69.2 (6.7)	0.424 (0.042)**
16 to 16.9	365	57.7 (8.7)**	166.7 (7.2)**	20.8 (2.9)**	70.6 (7.1)**	0.424 (0.045)**
17 to 17.9	252	60.8 (10.3)**	168.1 (7.4)**	21.5 (3.3)**	72.1 (7.4)**	0.430 (0.044)*
*Total*	*3460*	45.5 (13.0)*	151.9 (14.1)**	19.4 (3.3)**	66.0 (8.1)**	*0.436 (0.048)*
Girls						
9 to 9.9	308	32.1 (7.4)	134.6 (7.6)	17.6 (3.0)	59.3 (6.6)	0.441 (0.041)
10 to 10.9	659	35.0 (7.9)	138.4 (7.6)	18.1 (3.0)	60.9 (7.4)	0.439 (0.047)
11 to 11.9	645	38.3 (7.9)	143.7 (7.5)	18.4 (2.9)	62.1 (6.7)	0.432 (0.044)
12 to 12.9	549	42.8 (8.6)	148.5 (7.3)	19.3 (3.0)	63.2 (6.9)	0.426 (0.044)
13 to 13.9	472	47.4 (9.0)	152.4 (6.3)	20.3 (3.2)	65.2 (7.3)	0.427 (0.046)
14 to 14.9	609	51.0 (8.9)	154.6 (6.5)	21.3 (3.3)	67.3 (8.0)	0.436 (0.052)
15 to 15.9	504	52.7 (8.6)	155.7 (6.8)	21.7 (3.1)	68.5 (7.1)	0.440 (0.046)
16 to 16.9	450	53.9 (8.6)	156.4 (5.8)	22.0 (3.1)	68.7 (7.7)	0.440 (0.049)
17 to 17.9	296	55.1 (9.3)	156.8 (6.5)	22.4 (3.6)	69.5 (7.7)	0.444 (0.050)
*Total*	*4494*	*44.8 (11.5)*	*148.7 (10.1)*	*20.0 (3.5)*	*64.8 (8.0)*	*0.436 (0.047)*

Data values are reported as mean and standard deviation (SD)

Significant difference between boys and girls within the same age group: *P < 0.01, **P < 0.0001

**Table 2:** Smoothed age- and sex-specific percentile of WC (cm) for Colombian children and adolescents aged 9–17.9 years

	n	M	SD	P_3_	P_10_	P_25_	P_50_	P_75_	P_90_	P_97_
Boys										
9 to 9.9	258	60.8	6.7	51.2	54.0	56.2	59.4	64.3	69.9	75.4
10 to 10.9	466	61.7	8.0	51.6	53.5	56.7	60.3	65.9	72.4	79.0
11 to 11.9	445	63.5	7.6	53.4	56.0	58.1	62.0	67.0	75.5	82.1
12 to 12.9	404	64.7	7.5	54.0	56.5	60.0	63.3	69.0	75.7	83.3
13 to 13.9	401	65.8	7.7	54.6	58.5	61.2	64.6	69.3	75.3	85.5
14 to 14.9	443	67.1	7.1	56.4	60.0	62.5	65.7	70.6	76.7	86.2
15 to 15.9	426	69.2	6.7	59.1	62.0	64.5	68.1	72.1	78.3	86.1
16 to 16.9	365	70.6	7.1	59.5	63.2	66.6	70.0	74.0	78.6	87.7
17 to 17.9	252	72.1	7.4	61.0	64.7	67.6	71.2	75.4	82.3	88.7
*Total*	*3460*	*66.0*	*8.1*	*53.6*	*56.5*	*60.5*	*65.3*	*70.5*	*76.5*	*84.1*
Girls										
9 to 9.9	308	59.3	6.6	50.1	52.0	54.4	58.0	63.4	68.8	74.5
10 to 10.9	659	60.9	7.4	50.8	53.0	55.8	59.6	64.6	71.0	79.0
11 to 11.9	645	62.1	6.7	52.5	54.8	57.4	60.8	66.0	71.0	76.7
12 to 12.9	549	63.2	6.9	53.0	55.8	58.2	61.9	67.0	72.5	79.1
13 to 13.9	472	65.2	7.3	53.6	57.1	60.3	64.3	69.4	74.3	82.0
14 to 14.9	609	67.3	8.0	55.2	59.0	62.5	66.9	72.0	77.0	82.9
15 to 15.9	504	68.5	7.1	57.0	60.5	64.2	67.5	72.3	77.0	86.0
16 to 16.9	450	68.7	7.7	57.3	60.7	63.8	68.0	72.9	78.3	85.2
17 to 17.9	296	69.5	7.7	58.0	61.0	64.5	68.5	73.3	79.4	88.9
*Total*	*4494*	*64.8*	*8.0*	*52.2*	*55.5*	*59.0*	*64.0*	*69.5*	*75.1*	*81.9*

*M* mean, *SD* standard deviation, *P* percentile

**Table 3:** Smoothed age- and sex-specific percentile values of WHtR for Colombian children and adolescents aged 9–17.9 years

	n	M	SD	P_3_	P_10_	P_25_	P_50_	P_75_	P_90_	P_97_
Boys										
9 to 9.9	258	0.455	0.044	0.395	0.409	0.424	0.447	0.483	0.516	0.553
10 to 10.9	466	0.450	0.052	0.383	0.398	0.418	0.442	0.477	0.519	0.567
11 to 11.9	445	0.448	0.047	0.374	0.399	0.417	0.438	0.472	0.513	0.553
12 to 12.9	404	0.440	0.047	0.373	0.390	0.409	0.429	0.461	0.509	0.557
13 to 13.9	401	0.429	0.048	0.370	0.385	0.401	0.419	0.446	0.489	0.550
14 to 14.9	443	0.423	0.043	0.362	0.381	0.394	0.413	0.443	0.483	0.533
15 to 15.9	426	0.424	0.042	0.367	0.379	0.396	0.416	0.442	0.482	0.528
16 to 16.9	365	0.424	0.045	0.364	0.380	0.399	0.420	0.441	0.484	0.524
17 to 17.9	252	0.430	0.044	0.370	0.386	0.402	0.421	0.453	0.491	0.530
*Total*	*3460*	*0.436*	*0.048*	*0.370*	*0.388*	*0.404*	*0.427*	*0.457*	*0.501*	*0.546*
Girls										
9 to 9.9	308	0.441	0.041	0.378	0.394	0.413	0.435	0.465	0.495	0.541
10 to 10.9	659	0.439	0.047	0.373	0.390	0.408	0.429	0.463	0.501	0.549
11 to 11.9	645	0.432	0.044	0.370	0.386	0.402	0.427	0.456	0.491	0.531
12 to 12.9	549	0.426	0.044	0.360	0.376	0.395	0.421	0.450	0.484	0.532
13 to 13.9	472	0.427	0.046	0.352	0.376	0.398	0.424	0.452	0.482	0.537
14 to 14.9	609	0.436	0.052	0.364	0.385	0.403	0.430	0.464	0.501	0.549
15 to 15.9	504	0.440	0.046	0.366	0.387	0.411	0.435	0.468	0.498	0.538
16 to 16.9	450	0.440	0.049	0.363	0.388	0.410	0.434	0.467	0.497	0.552
17 to 17.9	296	0.444	0.050	0.371	0.389	0.410	0.434	0.473	0.512	0.562
*Total*	*4494*	*0.436*	*0.047*	*0.365*	*0.385*	*0.405*	*0.429*	*0.461*	*0.496*	*0.539*

*M* mean, *SD* standard deviation, *P* percentile

**Fig. 1:**
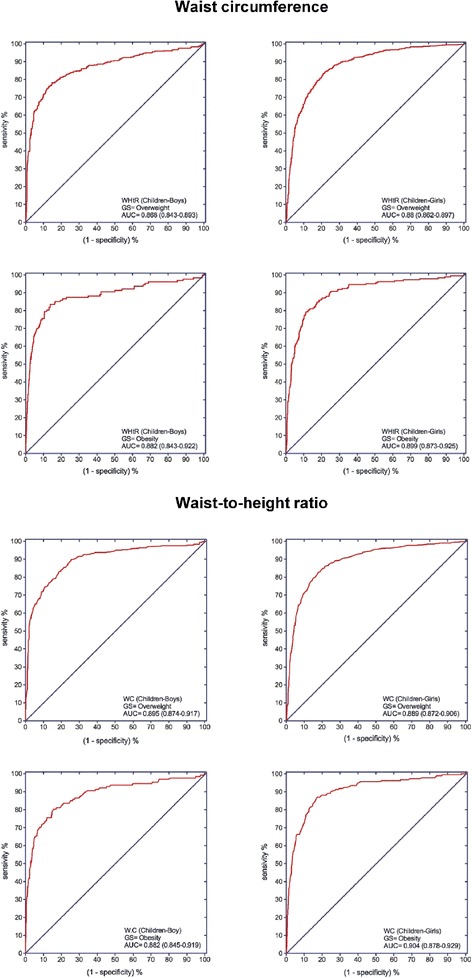
Receiver operating characteristic (ROC) curve for WC and WHtR to detect overweight (*top*) or obesity (*bottom*) according to the IOFT criteria for Colombian children aged 9.0–12.9 years. GS: Gold standard; AUC: Area under the curve (95% confidence interval)

**Fig. 2:**
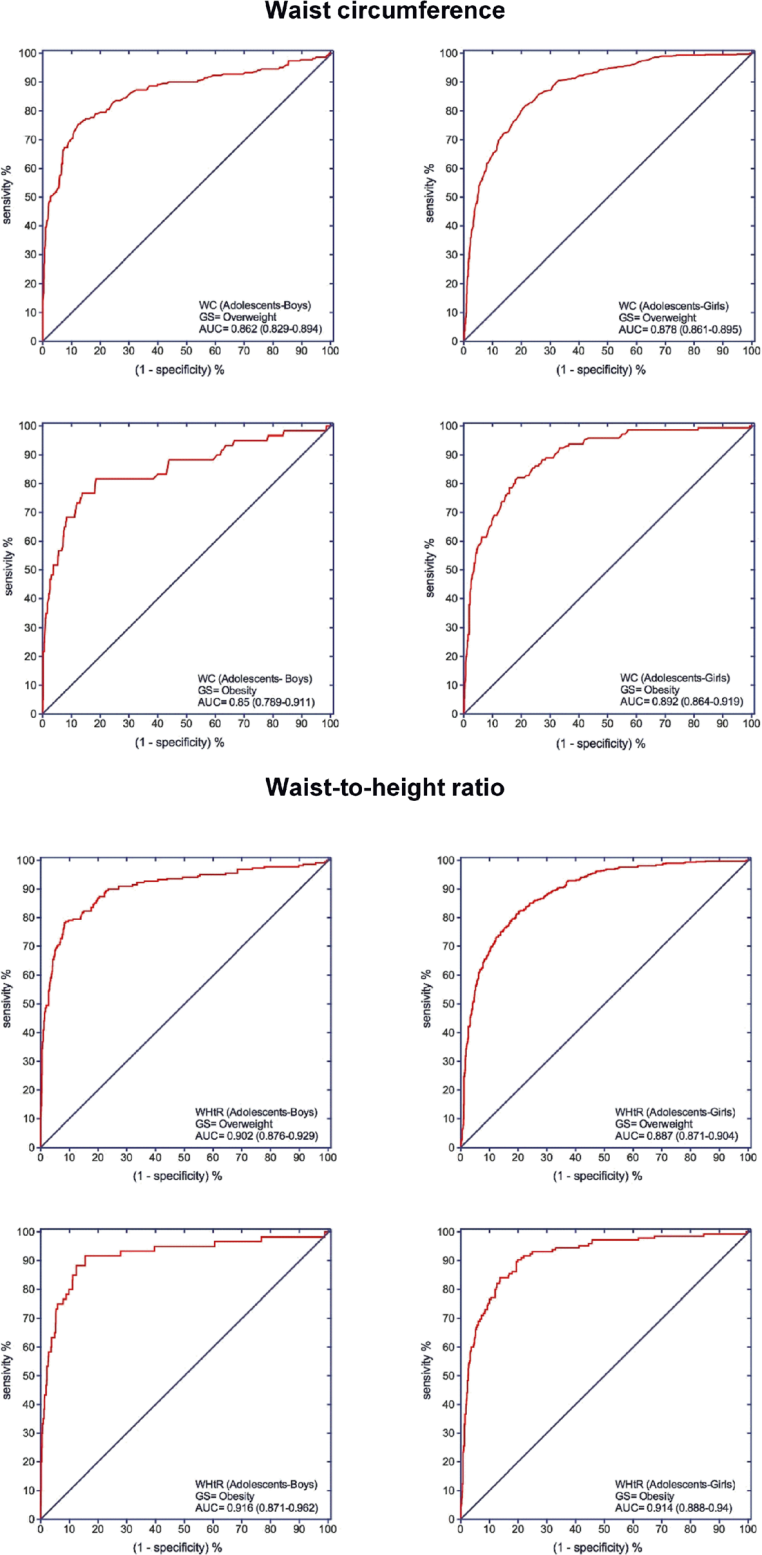
Receiver operating characteristic (ROC) curve for WC and WHtR to detect overweight (*top*) or obesity (*bottom*) according to the IOFT criteria for Colombian adolescents aged 13.0–17.9 years. GS: Gold standard; AUC: Area under the curve (95% confidence interval)

ROC analysis showed that both WC and WHtR had a high discriminating power to detect IOTF ovsserweight and obesity (Figs. 1 and 2). With respect to overweight among the boys in the study population, the cut-off point value of 62.7 cm for WC provided a sensitivity of 89.8%, a LR (+) value of 3.52, specificity of 74.5% and LR (−) value of 0.14. In girls, the cut-off point value of 62.8 cm for WC provided a sensitivity of 82.1%, a LR (+) value of 4.72, specificity of 82.6% and a LR (−) value of 0.22. For obesity in the boys, the cut-off value of 67.9 cm for WC provided a sensitivity of 87.0%, a LR (+) value of 5.06, specificity of 82.8% and a LR (−) value of 0.16. In the girls, the cut-off value of 65.9 cm for WC provided a sensitivity of 87.0%, a LR (+) value of 5.06, specificity of 82.8% and a LR (−) value of 0.16 (Fig. 1 and Table 4). The ROC curve for WHtR was also obtained, using a cut-off value of 0.459 (Fig. 2 and Table 4). For overweight, with this cutoff point, in the boys the sensitivity was 78.2%, the LR (+) 5.28, the specificity 85.2% and the LR (−) 0.26. In the girls, the cut-off value was 0.436, the sensitivity 84.6%, the LR (+) 3.83, the specificity 77.9% and the LR (−) 0.20. For obesity in the boys, the cut-off value of 0.485 was used, producing a sensitivity of 83.5%, LR (+) 6.05, specificity 86.2% and LR (−) 0.19. In the girls, the cut-off value was 0.472, producing a sensitivity of 79.3%, LR (+) 7.02, specificity 88.7% and LR (−) 0.23.

**Table 4:** Area under the receiver-operating characteristic curves (AUC) for WC and WHtR indices among Colombian children and adolescents aged 9–17.9 years

	WC				WHtR			
	Overweight		Obesity		Overweight		Obesity	
Children	Boys (9–12.9 y)	Girls (9–12.9 y)	Boys (9–12.9 y)	Girls (9–12.9 y)	Boys (9–12.9 y)	Girls (9–12.9 y)	Boys (9–12.9 y)	Girls (9–12.9 y)
AUC (95% CI)	0.895 (0.874–0.917)	0.889 (0.872–0.906)	0.882 (0.845–0.919)	0.904 (0.878–0.929)	0.868 (0.843–0.893)	0.881 (0.862–0.897)	0.882 (0.843–0.922)	0.899 (0.873–0.925)
Optimal cut-offs	62.7	62.8	67.9	65.9	0.459	0.436	0.485	0.472
J-Youden	0.643	0.647	0.647	0.698	0.634	0.625	0.697	0.68
Sensitivity (%)	89.8%	82.1%	80.3%	87.0%	78.2%	84.6%	83.5%	79.3%
Specificity (%)	74.5%	82.6%	84.4%	82.8%	85.2%	77.9%	86.2%	88.7%
LR (+)	3.52	4.72	5.15	5.06	5.28	3.83	6.05	7.02
LR (−)	0.14	0.22	0.23	0.16	0.26	0.20	0.19	0.23
	Overweight		Obesity		Overweight		Obesity	
Adolescents	Boys (13–17.9 y)	Girls (13–17.9 y)	Boys (13–17.9 y)	Girls (13–17.9 y)	Boys (13–17.9 y)	Girls (13–17.9 y)	Boys (13–17.9 y)	Girls (13–17.9 y)
AUC (95% CI)	0.862 (0.829–0.894)	0.878 (0.861–0.895)	0.850 (0.789–0.911)	0.892 (0.864–0.919)	0.902 (0.876–0.929)	0.887 (0.871–0.904)	0.916 (0.871–0.962)	0.914 (0.888–0.94)
Optimal cut-offs	73.8	68.9	74.1	76.6	0.458	0.445	0.463	0.468
J-Youden	0.630	0.605	0.631	0.550	0.699	0.619	0.762	0.704
Sensitivity (%)	75.5%	81.9%	81.7%	63.4%	78.6%	82.2%	91.7%	90.3%
Specificity (%)	87.5%	78.6%	81.4%	91.6%	91.3%	79.7%	84.5%	80.1%
LR (+)	6.04	3.83	4.39	7.55	9.03	4.05	5.92	4.54
LR (−)	0.28	0.23	0.22	0.40	0.23	0.22	0.10	0.12

*AUC* area under curve, *LR (+)* positive likelihood ratio, *LR (−)* negative likelihood ratio

## Discussion

This paper provides the first age and sex-specific WC and WHtR percentiles to be determined for Colombian children and adolescents (aged 9.0–17.9 years). These results can be used as a baseline for long-term health monitoring in rural and urban areas. The participants’ body weight increased with age, as was to be expected; moreover, this was in line with the findings of previous studies [34, 35]. In our sample, the girls aged 9.0–13.9 years had higher mean Ht values than the boys of the same age. According to Cousiminer et al. [36], this increase in Ht could be related to physical and sexual development, which generally occurs earlier in girls, as has been reported elsewhere [37, 38].

The boys and girls in our sample had similar mean BMI values, although the prevalence of overweight and obesity was higher among the girls, which corroborates previous findings [27, 39–41]. Following Hirschler et al. [23], who studied a population of indigenous children in Argentina, we also found (as expected) that mean WC values increased with age. Moreover, studies have shown that the distribution pattern of subcutaneous fat varies with age [41–44], with a tendency for fat to be deposited in the central area of the body instead of in peripheral areas, which heightens the risk of cardiovascular disease [43, 44].

Moreover, in line with previous studies [45, 46] our results found that for both males and females, mean WHtR values decreased with age, as was to be expected. In accordance with Wang et al. [47], in their study with children of Beijing (China), our results show a moderate correlation between WC and BMI in both boys and girls. Nonetheless, we agree with Smith and Haslam [48] that it would be useful to know which WC values are considered normal for each BMI level. This would permit corrective measures to be applied to persons with anomalous WC values, thus improving their cardiometabolic health. Our results also showed a positive correlation between WHtR and BMI, in both sexes, which is in line with the results of previous research [49, 50], according to which WHtR is an accurate indicator of cardiometabolic risk.

Regarding WC percentages for boys up to the age of 13.9 years, the 50th percentile presented higher values than those obtained by the same percentile of girls of the same age. This finding corroborates previous research using the LMS method [27]. After the age of 14 years, however, the girls in the 50th percentile had higher values than the boys. Differences between the sexes were less striking in the 97th percentile, although an upturn in WC values was observed in the boys aged 11.0–14.9 years, while no such variation occurred among the girls. Again, these results are indicative of sexual dimorphism in body composition [51].

Gender-related differences were also present in the WHtR: thus, boys aged 9.0–13.9 years had higher WHtR values than girls in the same age group. However, in the 14.0–17.9 year range, the tendency was reversed, and the girls had higher WHtR values in all percentiles. These results are in line with previous research on other sample populations [52, 53].

The ROC results showed that both WC and WHtR have a high discrimination power to detect overweight and obesity in our sample population of children and adolescents. The optimal cut-off value for WC among the boys with overweight was 73.8 cm. This exceeded the cut-off values for adolescent girls and for children of both sexes. These results are similar to those obtained in previous research [45, 54]. Concerning obesity, the highest cut-off point was obtained for adolescent girls (76.6 cm), which coincides with previous international studies [55, 56].

The ROC analysis for WHtR in the overweight category produced similar cut-off values for children and adolescents of both sexes. For the obesity category, the values were also similar, especially in obese adolescents of both sexes. However, these values were lower than the 0.50 that Bacopoulou et al. [53] established as a cut-off point for obesity in Greek adolescent boys and girls. WC and WHtR [8, 10] are known to be better predictors of cardiovascular disease risk in children than BMI. Indeed, prospective and case–control studies have shown that even with a normal BMI, persons with a low degree of physical fitness are at increased risk of cardiovascular disease and premature death [57, 58]. In Latin America, for example, Colombia has undergone rapid urbanisation and integration with world markets [20]. This has led to a worsening of dietary habits, coupled with declining levels of physical activity among the population, with negative consequences on body composition and overall health [20, 56, 59]. These changes are contributing to a global increase in the prevalence of non-communicable diseases [57, 60]. Therefore, the inclusion of WC and WHtR within health monitoring systems is justifiable and has been recommended [8, 10]. Schools may be an ideal setting in which to monitor levels of fitness [61] and to formulate and apply specific strategies to promote young people’s future health.

This study has certain limitations. First, it included participants from only one region in Colombia; therefore, caution is needed in extrapolating these findings to all Colombian children and adolescents. Second, we did not examine the potential impact of recognised determinants such as socio-economic factors, diet, patterns of physical activity and ethnic factors, which could modulate the growth and levels of body fat. Third, the study population was composed of young people at state-funded schools in a single city; therefore, the data obtained are not fully representative of the population of Bogotá or of Colombia. However, this city is the largest in the country, and is home to 15% of its population. It includes a mix of locally-born residents and those arriving from other regions, and so it is racially and culturally diverse. Another limitation is that the study did not evaluate students attending private schools. This restriction arose because the study was conducted in collaboration with the Bogotá District Education Department, which only has jurisdiction among public schools. However, 85% of school-age children are enrolled in the city’s public school system. Nevertheless, extrapolation of our findings to all children and adolescents in Bogotá or in Colombia requires caution. Future population-based studies incorporating data for nationally representative samples, such as the one recently conducted in Argentina, are still needed in Colombia and in other countries in the region [42–46]. This is an area in which further research is needed. However, the above-noted limitations do not invalidate the results obtained.

This study also has strengths that should be emphasised. The percentile values presented, based on a large, newly-compiled population sample, are the first to be obtained for WC and WHtR in Colombian children and adolescents. The results obtained enable us to make an accurate description of the anthropometric characteristics of the population studied and to highlight their age and gender-related variations. These percentiles can be used as a benchmark with which to compare the body composition of individuals of a corresponding age in the city, country and region.

## Conclusions

In conclusion, this is the first comprehensive study to present smoothed age and sex-specific WC and WHtR percentiles for Colombian children and adolescents aged 9.0–17.9 years. Both parameters present high sensitivity and specificity as predictors of overweight and obesity among the study population. The growth charts obtained will enable healthcare workers and researchers to diagnose and monitor children and adolescents, and could be used for the early detection of obesity.

## Abbreviations

AUC: Area under the receiver-operating characteristic curves
BMI: Body mass index
BW: Body weight
FUPRECOL: Asociación de la fuerza prensil con manifestaciones de riesgo cardiovascular tempranas en niños y adolescentes colombianos (*Association between grip strength and early signs of cardiovascular risk in Colombian children and adolescents*)
Ht: Height
IOTF: International Obesity Task Force
ISAK: International Society for the Advancement of Kinanthropometry
ROC: Receiver operating characteristics curve
SD: Standard deviation
TEM: Technical error of measurement
USA: United States of America
WC: Waist circumference
WHtR: Waist-to-height ratio
